# Controlled Degradation of PBAT for PBAT/PLA Blend Melt‐Blown Nonwovens

**DOI:** 10.1002/marc.202500276

**Published:** 2025-07-10

**Authors:** Gillian Binley, Tizazu H. Mekonnen

**Affiliations:** ^1^ Department of Chemical Engineering Institute of Polymer Research Waterloo Institute of Nanotechnology University of Waterloo Waterloo Ontario Canada

**Keywords:** hydrolysis, melt‐blowing, poly(butylene adipate‐co‐terephthalate), poly(lactic acid), rheology

## Abstract

This study utilized the chain‐scission capability of peroxides, such as aqueous hydrogen peroxide (H_2_O_2_), to induce controlled degradation of poly(butylene adipate‐co‐terephthalate)(PBAT) through reactive batch mixing with the objective of increasing the melt flow index (MFI). The effects of the peroxide concentration and processing time were examined, and the results showed that concentration had the greatest impact, with an approximate 450% increase in MFI at the optimal peroxide concentration. On the other hand, the peroxide treatment had a minimal impact on crystallinity and thermal properties. Degradation was deemed to occur chiefly via random chain scission with contributions from heat and hydrolysis, as supported by proton nuclear magnetic resonance spectroscopy (^+^HNMR). The treated PBAT sample showed promise in melt‐blown micro‐fiber production, producing fibers with a 68% smaller average diameter than that of the untreated PBAT. The treated PBAT was then blended with various levels of high MFI poly(lactic acid) (PLA) to optimize properties and cost of the resulting micro‐fiber material. As expected, the blends demonstrated increased tensile strength and decreased elongation at break with higher PLA contents, up to 30% and 13%, respectively, successfully balancing the material properties of the PBAT starting material. Despite these favorable tensile properties, the material blend remained suboptimal due to evidence of phase separation. To bridge this incompatibility, maleation was implemented, resulting in a polymer characterized by improved homogeneity, thereby enabling the production of uniform fibers without compromising desired tensile properties. The melt‐blowing generated PBAT‐PLA micro‐fibers can have applications as a sustainable alternative for polypropylene‐dominated HVAC air filters, medical masks, etc.

## Graphical abstract

Controlled degradation of PBAT for biodegradable melt‐blown nonwovens.

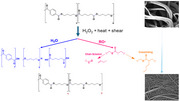

## 1 Introduction

Plastic is deeply enmeshed in our day‐to‐day lives, from car parts to coffee cups to clothing that has a limited life span. Regrettably, the majority of such plastics are nonbiodegradable, and stay hundreds of years in the ecosystems, while slowly shedding micro‐plastics to the air we breathe, drinking water, and arable land, with serious safety concerns [1]. In an attempt to maintain the convenience and reliability of plastic while reducing the environmental impact, researchers have turned to studying biodegradable plastics, such as poly(butylene adipate‐co‐terephthalate) (PBAT) and poly(lactic acid) (PLA) [2]. Unfortunately, these substitutes are relatively new and have limitations that are not present in the conventional polymers used today. One such limitation is the low melt flow index (MFI) of many of the available biodegradable options [3]. Low MFI can make plastics unsuitable for processing techniques requiring low melt viscosity, including melt spinning, melt blowing, and blow molding. While some high MFI varieties of PLA are available on the market, they are typically higher costs due to the specialized properties and applications. As such, the implementation of biodegradable polymers in these applications necessitates engineering a simple, cost‐effective solution for improved compatibility with these processes.

One method to modify the viscosity and MFI of polymers is through controlled degradation. Reducing the molecular weight of the polymer increases the flow by decreasing the chain entanglement [4]. There are several techniques that can be applied during polymer processing to induce controlled degradation, such as the introduction of enzymes, thermal treatment, and reactive extrusion [4, 5, 6]. Reactive extrusion frequently utilizes the chain scission capability of peroxides to promote degradation [7, 8]. Typically, organic peroxides are chosen; however, they are known to release undesirable volatile compounds (e.g., acetone and tert‐butanol in the case of 2,5‐dimethyl‐2,5‐di‐tert‐butylperoxyhexane (DHBP)) [7]. Hydrogen peroxide (H_2_O_2_), on the other hand, generates only water and oxygen as byproducts, making it a suitable option for a more environmentally friendly approach. H_2_O_2_ has proven effective for use in controlled degradation of several polymers, including polypropylene and chitosan [9, 10].

In our previous work, this technique was applied to PLA, where the molecular weight was successfully reduced by up to 70% and the MFI increased by up to 150 times [11]. In this work, we instead focus on the controlled degradation of PBAT, a biodegradable polymer with an even lower MFI than PLA [12]. While unmodified PLA had been previously melt‐blown [13, 14, 15], there are currently no studies focusing on melt‐blown PBAT as it is generally considered incompatible with the process. Therefore, this work aims to modify PBAT through aqueous H_2_O_2_‐induced controlled degradation to produce melt‐blown nonwovens.

The modified PBAT can then be blended with PLA to reduce costs and improve the final material. PBAT and PLA are known for having opposite weaknesses; while PLA is strong and brittle, PBAT is elastic and relatively weak [16]. There are many studies that blend the two polymers in order to get the best of both worlds [17, 18, 19]. A study by Yu et al. added modest amounts of PBAT (2%–10%) to PLA‐based nonwovens to improve elongation at break and enhance hydrophobicity, creating a material with an oil‐water separation efficiency of 96% [20]. In this study, along with several others, it was noted that phase separation occurs when the two polymers are combined in larger quantities (>10% of one with the other). [17, 18, 19, 20, 21] To combat the immiscibility and poor compatibility, compatibilization techniques like the use of epoxy oligomers, isocyanates, ionic liquids, and combinations of anhydrides and peroxides have been employed [16].

This study, therefore, examines the degradation effect of reactive batch mixing with aqueous H_2_O_2_ at different processing times and concentrations on PBAT. The study further looks at how this degradation impacts the compatibility of PBAT with the melt‐blowing process, both as an independent material and blended with various levels of PLA. Finally, maleation, grafting of maleic anhydride using peroxide, is examined as a compatibilization technique to improve blend miscibility.

## 2 Experimental

### 2.1 Materials

The Poly(butylene adipate‐co‐terephthalate) (PBAT) was provided by T&T Industry Group Ltd. (Shenzhen, China), and the Poly(lactic acid) (PLA), Luminy L105 [melt flow index (MFI) at 210°C, 70 g/10 min, T_melt_ 180–220°C], was obtained from TotalEnergies Corbion. Aqueous hydrogen peroxide (H_2_O_2_) (30 wt.%) was purchased from Sigma–Aldrich. Chloroform‐D (99.8%) was used as a solvent and purchased from Cambridge Isotope Laboratories.

### 2.2 Methods

#### 2.2.1 Reactive Batch Mixing

PBAT (∼150 g) was spray‐coated at room temperature with varied amounts of aqueous H_2_O_2_ and melt‐blended at 200°C in a HAAKE Polylab QC torque batch mixer at 60 rpm for 5–10 min. The processed samples were allowed to stabilize for at least 24 h at room temperature before characterization commenced. The concentration of H_2_O_2_ and processing times are described in Table 1. The degradation effects of heat and water were accounted for by Controls 1 and 2, respectively. Additional sample parameters were chosen to align with the previous study by Binley et al. [11].

**TABLE 1:** Sample identification and formulation of treated PBAT samples.

Sample ID	H_2_O_2_ Concentration (phr)	Processing time (min)
PBAT	0	0
Control 1	0	10
Control 2	0 (10 phr Water)	10
PBAT_5_5	5	5
PBAT_5_10	5	10
PBAT_7.5_7.5	7.5	7.5
PBAT_10_5	10	5
PBAT_10_10	10	10

#### 2.2.2 Proton Nuclear Magnetic Resonance (H NMR) Spectroscopy

PBAT samples were dissolved at room temperature in deuterated chloroform to ∼5 mg/mL. A Bruker 300 MHz high‐resolution nuclear magnetic resonance spectrometer was then used to obtain Proton (^1^H) spectra with 64 scans and a relaxation delay of 10 s.

#### 2.2.3 Melt Flow Index (MFI)

Using a Kayeness D4002 melt flow indexer, the MFI was measured in accordance with ASTM D1238 at 190°C with a load of 2.16 kg and a standard 1 mm bore. A total of three measurements were taken per sample.

#### 2.2.4 Compression Molding

A Carver hydraulic press was used to compression mold samples into compatible shapes for testing. H_2_O_2_‐treated PBAT samples were molded between two compressed hot plates at 180°C under six metric tons of pressure for 3 min to produce rheology disks of 1 mm in thickness and 25 mm in diameter. PBAT – PLA blend samples were also compression molded under the same conditions to produce flat sheets (76 × 75 × 0.2 mm^3^) for use in atomic force microscopy (AFM) and polarized optical microscopy (POM).

#### 2.2.5 Parallel‐Plate Rheology

Disks were prepared as previously described, and measurements were conducted using a HAAKE MARS rheometer. A shear strain of 1% was selected based on the linear viscoelastic region of PBAT. The rheological properties were evaluated using a frequency sweep from 0.1 to 100 rad/s at 200°C with a 1 mm gap under an ambient lab environment.

#### 2.2.6 Differential Scanning Calorimetry (DSC)

A DSC Q2000 (TA Instruments) was used to perform thermal analysis. Peroxide‐treated samples of approximately 6 mg were placed in separate aluminum T_zero_ pans. Testing was then conducted under a nitrogen flow rate of 50 mL/min. The samples were heated at a rate of 10°C/min to 200°C and held isothermal for 3 min after which they were cooled to −60°C at the same rate. Finally, the temperature was raised once again to 200°C at 10°C/min. The second heating cycle was used to determine glass transition temperature (T_g_), melting temperature (T_m_), crystallization temperature (T_c_), and melting enthalpy (H_mPBAT_) to avoid any thermal histories within the systems. Percent crystallinity (%χ_CrPBAT_) was calculated using Equation 1, where H^0^_mPBAT_, the heat of fusion value if PBAT were 100% crystalline, is 114 J/g [22].

(1) $$\%\;\chi_{CrPBAT}=\left(\frac{H_{mPBAT}}{H_{mPBAT}^{0}}\right)\;*100\%$$

PBAT/PLA blends were also evaluated in the same manner, except at a rate of 20°C/min to improve the detection of T_g._ Once again, the reported values, including melting enthalpy of PLA (H_mPLA)_ and cold crystallization enthalpy (H_ccPLA_), were taken from the second heating cycle, and percent crystallinity of the blends (%χ_CrBlend_) was calculated using Equation 2, where X_PBAT_ and X_PLA_ are the mass fractions of PBAT and PLA, respectively, and H^0^_mPLA_, the heat of fusion if PLA were 100% crystalline, is 93.7 J/g [23].

(2) $$\begin{array}{cc} \%\;\chi_{CrBlend} & =\left(X_{PBAT}\left(\frac{H_{mPBAT}}{H_{mPBAT}^{0}}\right)+X_{PLA}\left(\frac{H_{mPLA}-H_{ccPLA}}{H_{mPLA}^{0}}\right)\right)\\ & \;*100\% \end{array}$$

#### 2.2.7 Melt Blowing

Finely ground PBAT‐10‐5 and PLA were combined in various ratios (Table 2) prior to processing in a twin screw extruder (Process 11 Parallel Twin‐Screw Extruder, Thermofisher Inc). The compatibilized sample mixture (50‐50‐M) included 0.2 wt% dicumyl peroxide and 4 wt.% maleic anhydride (MA) in addition to the two polymers. The extruder was equipped with a custom die for melt‐blowing comprised of a jet angle cap, melt feed nozzle, and gas feed resembling Exxon slot dies commonly used in commercial melt‐blown fiber production [24, 25, 26]. The outer cap featured a single 2.5 mm diameter outer hole and a 128° impinging air jet angle to create blown fibers with heated nitrogen gas. The temperature of the nitrogen air jet was controlled using a transformer (Powerstat Variable Transformer) connected to a 750 W heating torch (Laramy Products, Lyndonville, VT, USA) and set to ∼350°C, and the gas volumetric flow rate was adjusted to 0.5 L/min using the nitrogen gas gage pressure. The extruder temperature and screw speed were set to 220°C and 100 rpm, respectively. A parchment paper‐covered conveyor belt rotating at 60 rpm was placed ∼40 cm away from the die opening to collect the fibers and produce nonwoven mats. Additional description of the experimental setup is found in Lee et al. [11, 13, 27].

**TABLE 2:** Sample code and formulation of PBAT/PLA melt‐blown fibers.

Sample ID	PBAT content (wt.%)	PLA content (wt.%)
70_30	70	30
50_50	50	50
50_50_M	50	50
30_70	30	70
PLA	0	100

#### 2.2.8 Polarized Optical Microscopy (POM)

The fiber distributions and crystal structures of the melt‐blown fabrics were investigated using an Olympus BX53M POM optical microscope equipped with a Linkam heat stage accessory and a polarized light filter. Fiber mat samples remained at ambient temperature and were placed between glass slides to capture images at 10x magnification. Using the Olympus Stream Basic software measuring tool, 95–105 fibers per sample were individually measured to determine fiber uniformity. The crystal structure was observed using thin film samples that were heated to 200°C at a rate of 10°C. Upon cooling, images were captured using a 20x objective lens.

#### 2.2.9 Scanning Electron Microscopy (SEM)

A Phenom Desktop SEM (Thermofisher Scientific, USA) was used to examine the surface morphology of the fiber mat samples at 500× magnification.

#### 2.2.10 Atomic Force Microscopy (AFM)

The homogeneity and surface morphology of PBAT and PLA blends were examined using AFM (Veeco Digital Instrument, Dimension 3100) with a NanoScope IV controller. Images were captured at a size of 10 × 10 µm2 using a scan rate of 1 Hz. To detect differences in stiffness of the two polymers in the blends, the tapping phase mode was used.

#### 2.2.11 Tensile

Tensile testing was conducted using a Shimadzu AGS‐X (Shimadzu Corp, Kyoto, Japan) tensile testing instrument equipped with a 2 kN load cell at 100 mm/min. Rectangular samples were cut from the center of the nonwoven mats and measured on average 25 mm in width, 100 mm in length, and 1 mm in thickness. A total of 5 samples were tested per processing condition.

## 3 Results and Discussion

### 3.1 Controlled Degradation of Poly(butylene adipate‐co‐terephthalate) (PBAT)

#### 3.1.1 Rheology and MFI

**FIGURE 1:**
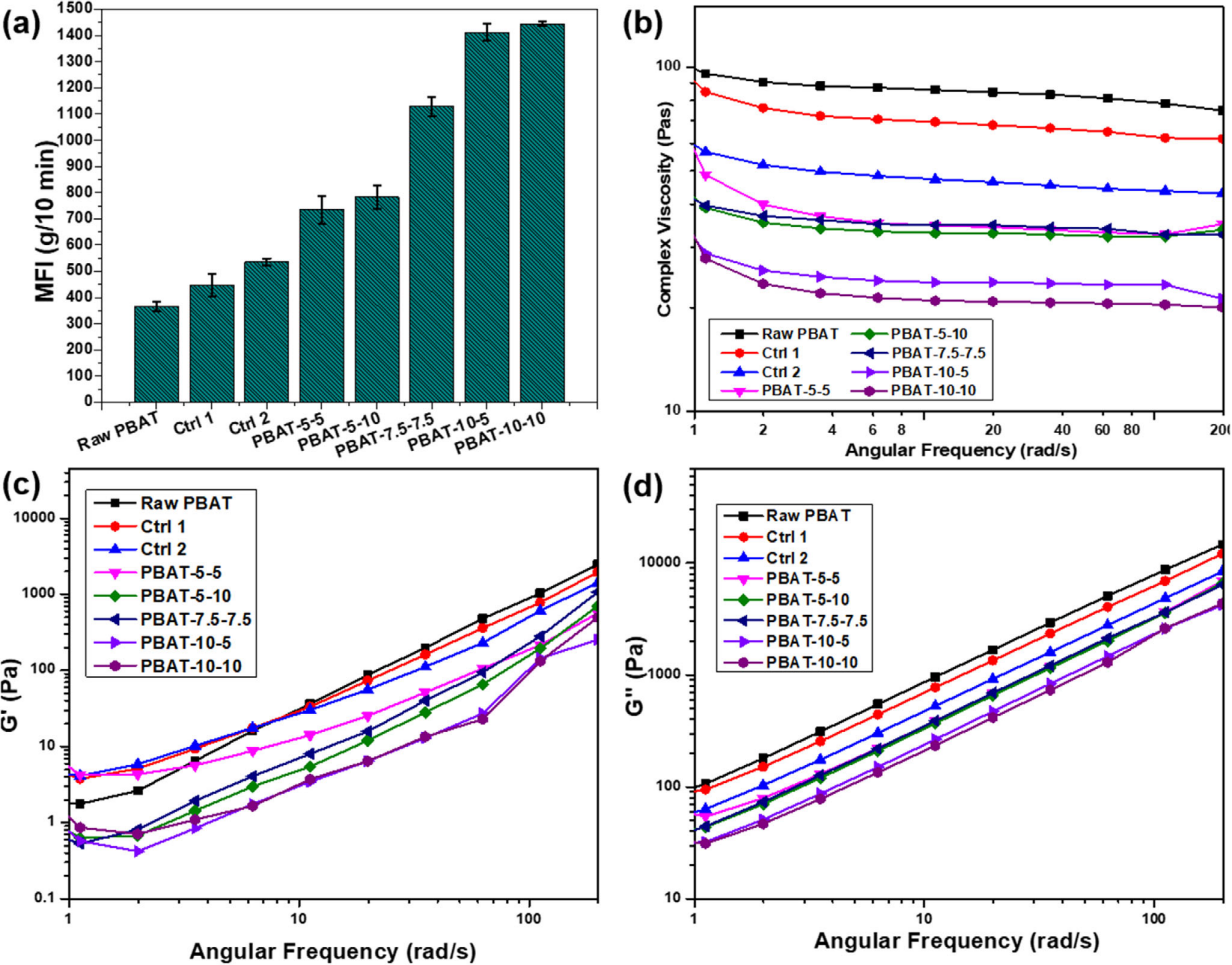
MFI (a), complex viscosity (b), storage modulus (c), and loss modulus (d) of hydrogen‐peroxide treated PBAT.

To get the complete picture of the changes to the melt flow behavior of the H_2_O_2_‐treated PBAT samples, both MFI testing and parallel plate rheology were performed (Figure 1). Both tests revealed the same pattern among samples with MFI increasing (i.e., viscosity decreasing) with both processing time and peroxide concentration. While the control samples show that the presence of heat and water is contributing to the overall effect, they are only contributing a small amount, with all treated samples having MFIs at least 36% greater than that of Control 2. This increase in MFI and decrease in viscosity is indicative of molecular weight reduction. This is further supported by the steep drop in viscosity at lower frequencies exhibited by the treated samples, especially PBAT‐5‐5, compared to the relative plateau found in the control samples. This can be attributed to the lower molecular weight of these samples, as the shorter chains relax more quickly and, as such, do not resist flow [6, 28]. While both processing parameters appear to enhance the degradative effect, H_2_O_2_ concentration demonstrates a much greater impact than processing time. MFI results were statistically analyzed using a two‐factor ANOVA to confirm the significance of each process parameter. As anticipated, H_2_O_2_ concentration has an incredibly low P‐value, while processing time and the interaction between the two have high values, deeming them statistically insignificant. As such, it can be stated that H_2_O_2_ concentration is the driving force behind the increase.

Interestingly, unlike with PLA [11], no crosslinking appears to be taking place despite the well‐established crosslinking effect of peroxides [29, 30, 31, 32]. PBAT has been shown to crosslink in the presence of peroxides like benzoyl peroxide [33] and di(tert‐butylperoxyisopropyl) benzene, with increased effect if a co‐agent, such as triallyl isocyanurate, is included in the reaction [32]. Considering the relatively high concentrations of peroxide used, it is possible that this crosslinking has begun to occur during the longer processing times if the maximum MFI was achieved between the points captured. This would cause the MFI values of the different processing times to be similar, as the crosslinking would reverse the molecular weight reduction, restoring the original level of entanglement. If MFI were to decrease at much longer processing times, then it would be possible to confirm; however, at this time, there is insufficient data to declare that crosslinking is occurring.

In addition to MFI measurement, parallel plate rheology analysis was used to observe the impact of the peroxide concentration and processing time on the complex viscosity, storage, and loss moduli of the treated samples (Figure 1b‐d). Like with the complex viscosity, no change to the overall behavior was shown, only a reduction among the treated samples. Once again, the greatest reduction was associated with the increase in the H_2_O_2_ concentration, while processing time had little impact. For instance, samples PBAT 5‐5, PBAT 10‐10, and PBAT 10–5 show an almost plateau behavior at low frequencies (Figure 1b), indicating rate‐independent viscosity behavior, potentially due to narrow molecular weight distribution. The decrease in these parameters further indicates molecular weight reduction as entanglement would be reduced, resulting in a lower capacity for deformation. Further indication would be a shift in the crossover of storage and loss moduli; however, this did not occur within the testing range for neither treated nor untreated samples.

#### 3.1.2 Differential Scanning Calorimetry (DSC)

**FIGURE 2:**
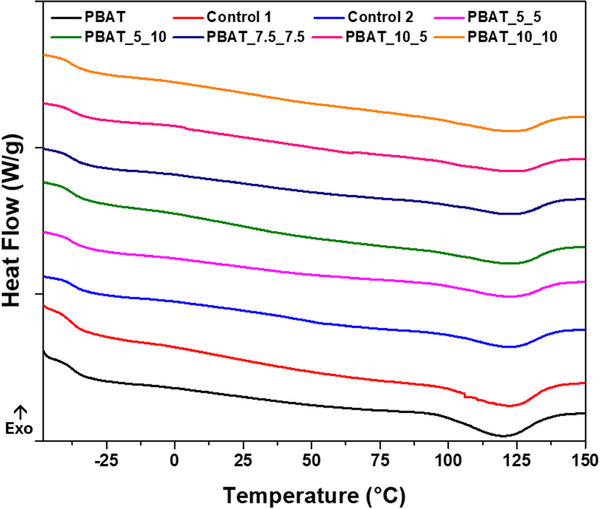
DSC thermograms of the second heating cycle (10°C/min) of H_2_O_2_‐treated PBAT samples.

Thermal analysis was performed using DSC to gain insight into changes to glass transition temperature (T_g_) and melting point (T_m_), as well as crystallinity (Figure 2) of the samples. H_2_O_2_ treatment appears to have had little impact on the thermal behavior of PBAT. While there is a slight decrease in T_g_ and an increase in T_m_, these changes are also present in the control samples and therefore can be attributed primarily to the heat and moisture impacts during the treatment. Crystallinity, however, does appear to be minimally impacted at higher peroxide concentrations and processing times, with the highest concentration processed for the longest time demonstrating a notable decrease in crystallinity. Interestingly, the highest crystallinity value, nearly equivalent to the untreated PBAT, is achieved in the PBAT_10_5 sample. This may suggest the possibility of chemi‐crystallization, an increase in crystallinity as a result of chain scission to the amorphous regions of a semicrystalline polymer, which was occurring prior to the 29% reduction during the additional processing time. [34]. As indicated by the rheology data, the higher peroxide concentration experiences significantly more molecular weight reduction (i.e., more chain scission); thus, it is more likely for chemi‐crystallinity to occur as chain scission occurs more readily in amorphous regions [35]. Overall, however, all changes observed during DSC testing were quite minimal, suggesting the impact of the treatment is insignificant (Table 3).

**TABLE 3:** Glass transition, melting temperatures, enthalpy of melting, and percent crystallinity of treated PBAT samples.

Sample ID	Tg (°C)	Tm (°C)	ΔHm (J/g)	Crystallinity (%)
PBAT	−36.75	119.86	12.45	10.9%
Control 1	−38.54	121.52	10.34	9.1%
Control 2	−37.80	122.57	10.57	9.3%
PBAT_5_5	−38.26	122.32	10.36	9.1%
PBAT_5_10	−37.85	122.69	9.79	8.6%
PBAT_7.5_7.5	−37.62	122.60	11.07	9.7%
PBAT_10_5	−37.66	121.78	12.68	11.1%
PBAT_10_10	−37.46	123.70	9.03	7.9%

#### 3.1.3 1H‐NMR

**FIGURE 3:**
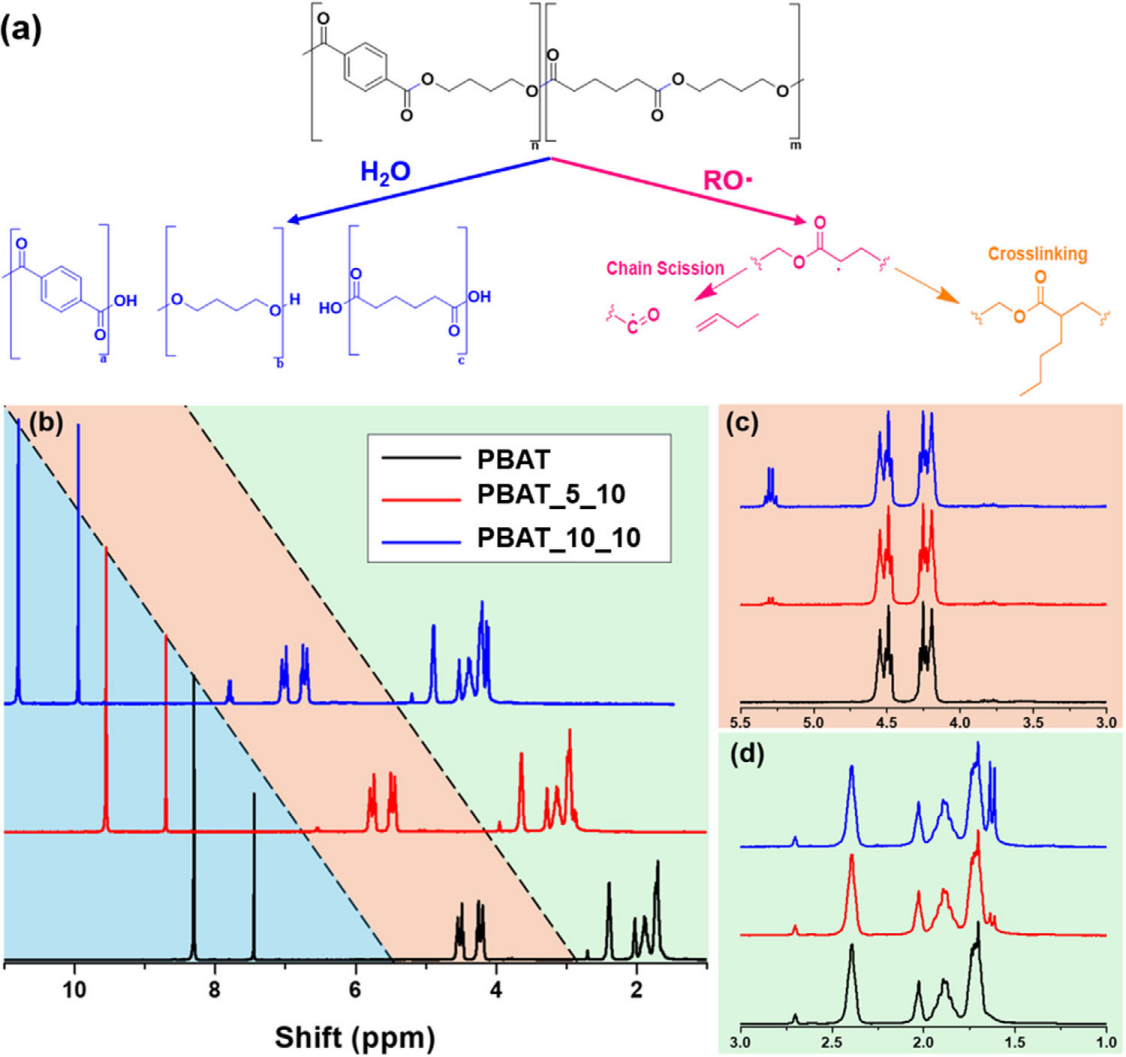
Proposed mechanism for reactions occurring during reactive batch mixing (a); 1‐H NMR spectrum (b) of treated PBAT samples with focus on the (c) terephthalic acid region and (d) butylene region.

Proton NMR results are shown in Figure 3, where it is clear that the peroxide treatment has impacted the structure of the treated PBAT. Two new peaks developed in the samples that had undergone reactive batch mixing: one at 5.15 and the other at 1.45 ppm. The size of these peaks increased with H_2_O_2_ concentration, suggesting the peroxide is at least partially responsible for the observed changes. The peak at 5.15 (Figure 3c) may be caused by an increase in terephthalic acid generation. When PBAT undergoes hydrolysis, chain scission occurs at the ester linkages, producing 1,4‐butanediol, adipic acid, and terephthalic acid [36, 37]. The presence of additional terephthalic acid, therefore, suggests bond cleavage at the ester bonds with contributions from the peroxide as well as the water in the solution.

The other peak of interest at 1.45 ppm is likely attributed to an increase in butylene‐based fragments and oligomers. The carbon of the methyl group near the C═O group is an ideal site for an attack by the free radical from the peroxide, which can then develop into a radical that is capable of both chain scission and crosslinking. [38] From the rheology data, it was clear that in the studied system, chain scission is dominant, and the impact of crosslinking, if any, is limited. This aligns with La Mantia et al., who established that chain scission dominates the free radical reaction with PBAT under wet conditions, such as those present with aqueous H_2_O_2_ [38]. When the radical reaction proceeds down the route of chain scission, butylene fragments are a byproduct, therefore aligning with the new peak. Since this new peak is associated with free radical degradation, it is clear that the peroxide, and not simply the heat and water, is responsible for the observed degradation. This is also emphasized by the intensity of the peroxide‐induced peak, being much greater than that at 1.45 ppm, which was occurring due to the moisture and peroxide jointly. All proposed reactions that are thought to occur during reactive batch mixing of PBAT with aqueous H_2_O_2_ are shown in Figure 3d. Overall, the proton NMR results align well with other evidence of degradation and support the proposed reaction mechanisms shown in Figure 3a.

#### 3.1.4 Melt‐Blown Fibers

**FIGURE 4:**
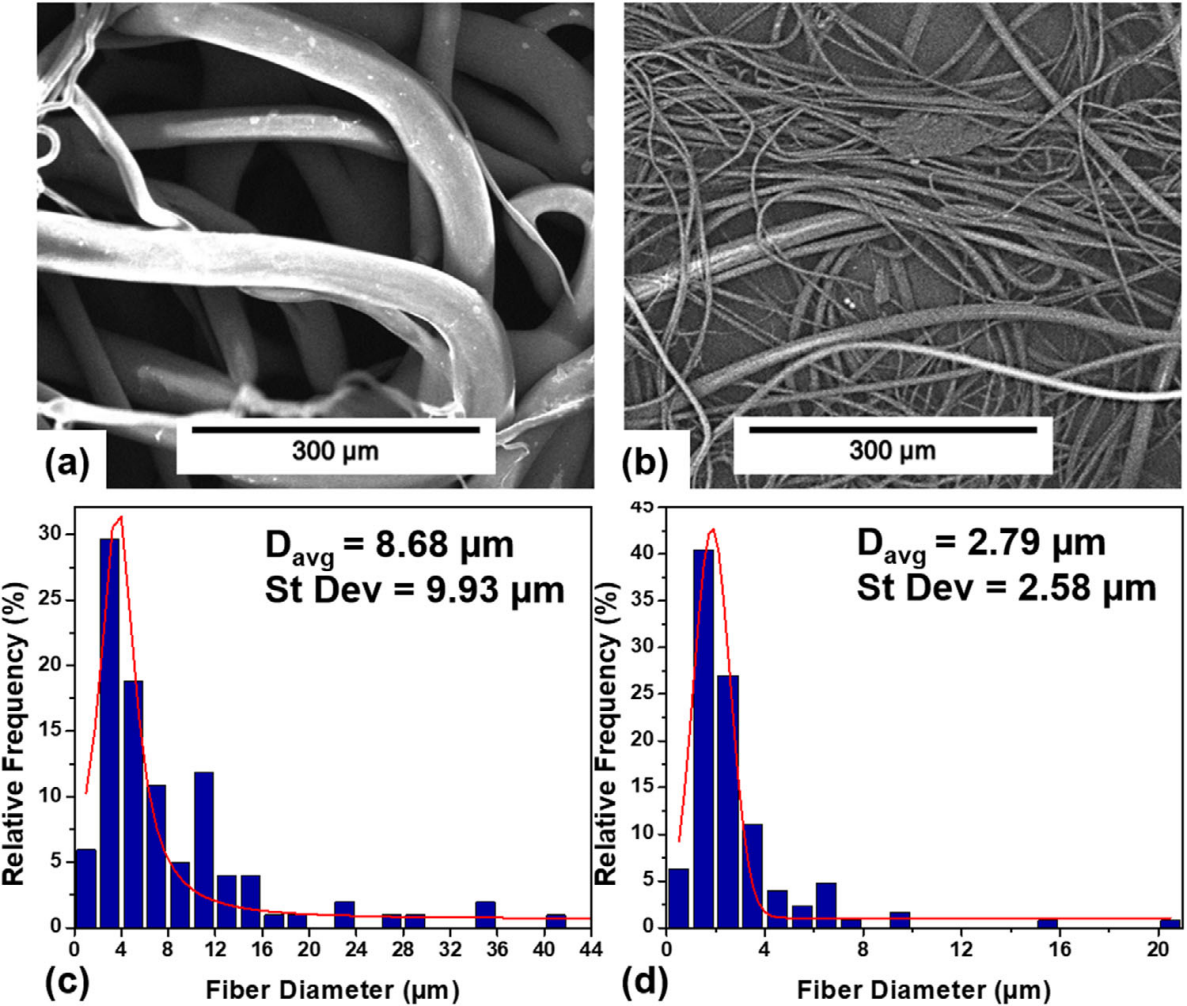
SEM images of melt‐blown PBAT (a) and PBAT‐10‐5 (b) at 500X magnification. As well as fiber diameter distribution of PBAT (c) and PABT‐10‐5 (d).

PBAT_10_5 was selected to proceed to melt blowing as the higher peroxide concentration had a much greater effect, while further processing beyond 5 min had very little additional gain. Untreated PBAT was also melt‐blown under the same conditions for comparison. SEM images and fiber distribution of both materials can be seen in Figure 4. The viscosity reduction associated with the peroxide treatment had a profound effect on both fiber diameter and distribution, reducing the average fiber diameter by 68% with the largest fibers of the treated sample being half the size of those of the untreated sample. Finer fibers are advantageous because they increase overall surface area, therefore improving filtration and absorption efficiency in practical applications [39]. The improved fiber uniformity is also beneficial because it allows for more predictable and better‐controlled pore sizes as well as improved mechanical properties since forces are distributed more evenly across the individual strands of material [40]. Further indication that the degraded sample produces improved non‐wovens is the presence of flies in the untreated PBAT sample. Flies, excessively thin frizzy fibers caused by fiber breakage, are commonly caused by an extreme difference between air and fiber flow speed [41]. Since the untreated PBAT is highly viscous, the air flow would be much faster in comparison, resulting in premature fiber breakage. The reduced viscosity of the treated sample, however, increases the speed of material flow and therefore prevents this from occurring. Overall, the peroxide treatment shows great promise in developing a controlled rheology grade of PBAT suitable for the melt‐blowing process.

### 3.2 Blending With PLA

#### 3.2.1 Atomic Force Microscopy

**FIGURE 5:**
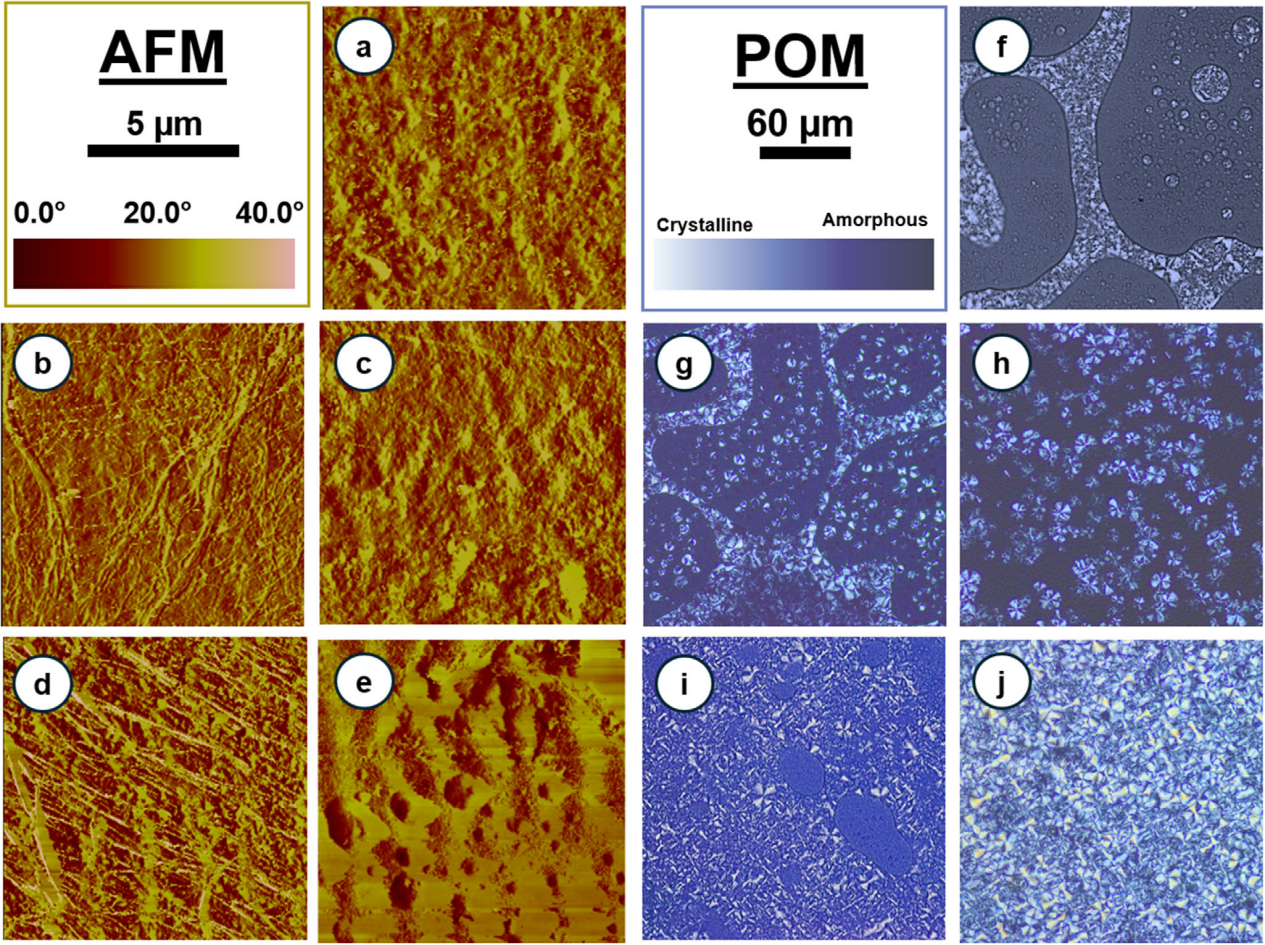
AFM images of 70_30 (a), 50_50 (b), 50_50_M (c), 30_70 (d), and PLA (e); POM images of the same blends in the same order (f‐j).

Atomic force microscopy (AFM) was used to gauge miscibility of the blends and identify phase separation through differences in the tapping hardness of the materials (Figure 5). AFM reveals a dark, bumpy surface for PBAT and a smoother, lighter surface for PLA. Therefore, the lighter spots and lines appearing in greater amounts with increasing PLA content are indicative of isolated PLA domains, suggesting phase separation. Figure 5b,c can be used to assess the effectiveness of maleation as a compatibilization strategy. Despite both samples being 50–50 blends of PBAT and PLA, the compatibilized sample shows little indication of phase separation while the untreated sample shows many distinct PLA fibers. This stark contrast emphasizes the value of a compatibilization agent in creating a homogenous mixture of PBAT and PLA. Overall, AFM reveals a clear distinction between blends and confirms the need for compatibilization.

#### 3.2.2 Polarized Optical Microscope (POM)

POM was also used to observe phase separation as well as investigate changes to the crystalline structure of the PBAT/PLA blends. PBAT is highly amorphous while PLA is highly crystalline; thus, the two polymers look distinctly different when observed under a polarized microscope. When blended together with no assistance from a compatibilizer, it is clear that phase separation occurs with the amorphous PBAT resting on top of the crystalline PLA (Figure 5). This aligns with the fact that PBAT is often slightly less dense than PLA, causing it to rise to the top while PLA sinks to the bottom [42]. Once a compatibilizer is added, however, only a single semi‐crystalline phase is visible (Figure 5h). While the blend more closely resembles PLA, it is clear that the sample selected is not solely PLA, as the crystallinity is much lower than that of the pure PLA. Additionally, the blend appears more similar to PBAT in the AFM image. Together, these two forms of microscopy confirm a successful compatibilized blend of the two and emphasize the impact of compatibilization in preventing phase separation.

#### 3.2.3 Differential Scanning Calorimetry (DSC)

**FIGURE 6:**
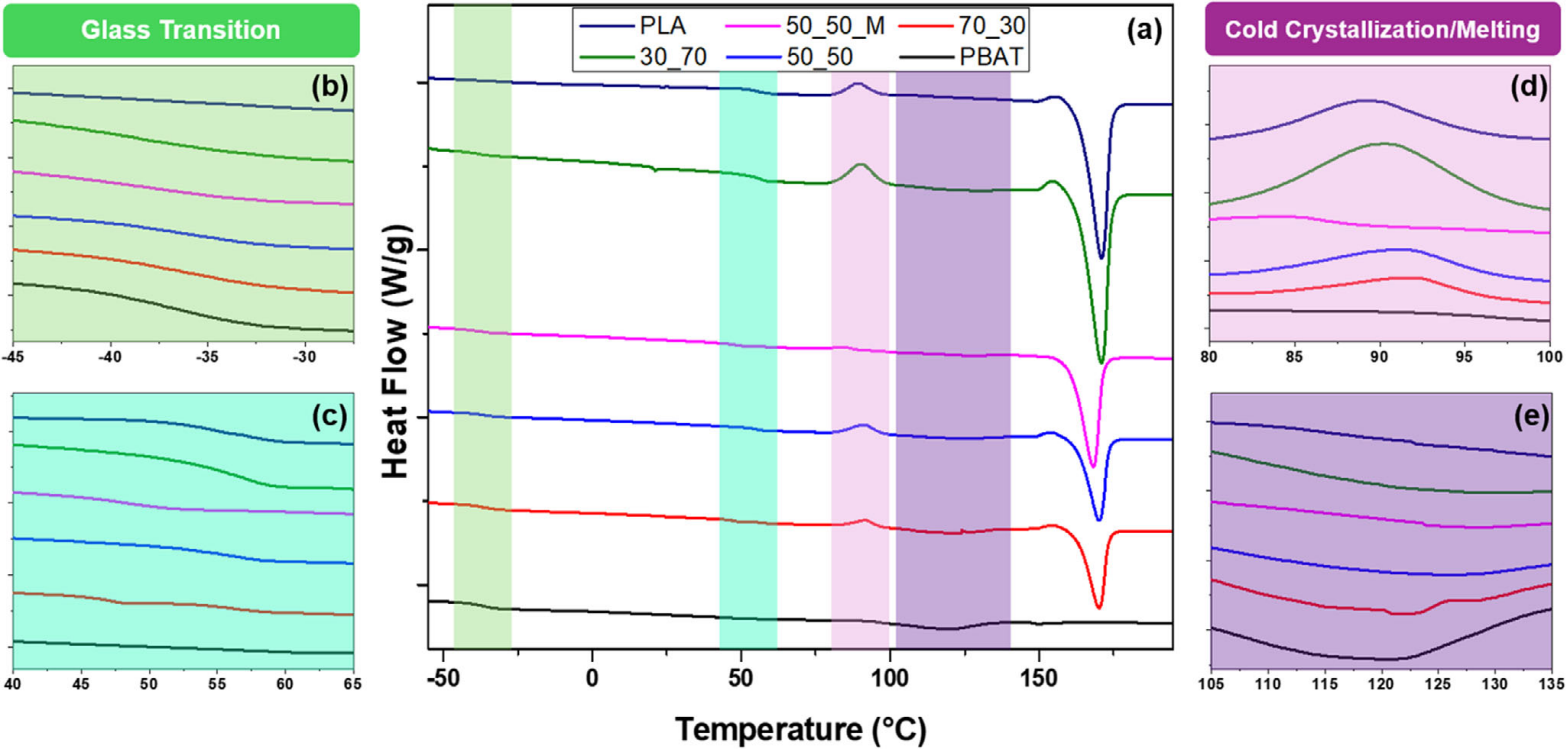
DSC thermogram of PBAT/PLA blends (a) with emphasis on the glass transition regions of PBAT (b) and PLA (c), as well as the cold crystallization region of PLA (d) and the melting region of PBAT (e).

DSC was performed to further analyze the thermal behavior and homogeneity of the blends (Figure 6). The blends, with the exception of the compatibilized sample, show discrete thermal events associated with each individual polymer. PBAT is represented by the glass transition around −36°C and the melting peak near 120°C, while PLA is responsible for the glass transition around 50°C, the melting peak at about 170°C, the cold crystallization peak around 90°C, and finally the small exothermic shoulder present just before the melting peak. Assigning these events to their respective polymers allows for the calculation of crystallinity based on the composition. Key thermal events and overall crystallinity are shown in Table 4. The maleated sample, however, is less easily split into its individual components. While both glass transition temperatures can be seen, only one melting peak is present, and the cold crystallization peak, while present, is nearly undetectable. The shoulder just before the melting peak has also vanished. As such, it can be stated that while there is still a small amount of separation detected, the majority of the specimen is a new material that behaves differently than either starting material. Both T_g_ values present in the compatibilized sample are reduced compared to their baseline. Since T_g_ is known to decrease with molecular weight, this is likely due to additional degradation caused by the peroxide present during the maleation process. The single melting peak is much higher in temperature than the melting peak of PBAT, but distinctly lower than that of PLA, further indicating the improved interaction between the two materials. Finally, the near disappearance and reduced temperature of the cold crystallization peak provide additional evidence of compatibilization [43].

**TABLE 4:** Glass transition, melting, and cold crystallization temperatures of PBAT/PLA blends as well as their enthalpies of melting and cold crystallization with calculated crystallinity.

Sample ID	T_g1_ (°C)	T_g2_ (°C)	T_m1_ (°C)	T_m2_ (°C)	T_cc_ (°C)	ΔH_m1_ (J/g)	ΔH_m2_ (J/g)	ΔH_cc_ (J/g)	Crystallinity (%)
**PBAT_10_5**	−36.07	—	118.75	—	—	10.36	—	—	9%
**70_30**	−34.80	47.18	121.87	169.86	92.42	19.39	2.96	3.50	7%
**50_50**	−36.17	51.62	124.16	169.83	91.89	1.97	25.05	5.94	11%
**50_50_M**	−39.61	47.99	—	167.63	85.56	—	29.88	0.54	16%
**30_70**	−38.74	52.03	120.66	170.11	90.55	0.67	35.31	8.33	20%
**PLA**	—	54.96	—	170.28	89.40	—	46.25	6.66	42%

The overall crystallinity of untreated samples, as calculated from DSC, also highlights the presence of phase separation. PBAT hinders the ability of PLA to crystallize, so even when PLA is in the majority (e.g., 30_70), the crystallinity is substantially reduced compared to pure PLA. Since PBAT has a lower melting point than PLA and the blend, PBAT remains in a molten state as the PLA begins to crystallize. Where this molten PBAT is present, the crystallization process may be impeded as it is more difficult for the crystals to align in an orderly fashion. Thus, the increase in the degree of crystallization of 50_50_M compared to 50_50 supports the claim of homogeneity since, by evenly dispersing the two materials within each other, nucleation sites may grow without interference from aggregated PBAT [44]. Overall, it is clear from DSC analysis, along with AFM and POM images, that maleation sufficiently mitigates phase separation within PBAT/PLA blends, resulting in a compatibilized material with balanced thermal and crystalline properties. Since a small amount of separation remains, further study of the amount and type of compatibilizer used would be required to optimize the process and improve crystal perfection.

### 3.3 Melt Blowing of PBAT/PLA Blends

#### 3.3.1 Fibers

Melt‐blown blends of the degraded PBAT sample and PLA were investigated using POM to identify any changes to the fiber diameter. As seen in Table 5, the average fiber diameter of all the blend ratios is nearly identical, with a similar distribution as well. The blends, however, show a notable increase in diameter compared to the starting materials, with the average being 15 and 20% greater than PBAT_10_5 and PLA, respectively. This increase in fiber size is likely due to the incompatibility of the materials, causing phase separation and inconsistent flow as indicated by DSC and microscopic analysis [40]. Additional evidence of phase separation is the presence of droplets and spots (Figure 6a). These malformations appear as larger blobs of material that are the result of surface tension instability [40]. While improving the flow of PBAT may improve its compatibility with PLA by bringing the viscosities closer together, it is insufficient for creating a completely homogenous blend. As such, the resulting untreated blends are not quite as compatible with the melt‐blowing process as the individual starting materials.

**TABLE 5:** Fiber distribution of PBAT/PLA blends.

Sample ID	Average fiber diameter (µm)	Standard deviation (µm)
PBAT_10__5	2.79	2.58
70_30	3.27	2.40
50_50	3.20	2.79
50_50_M	2.10	1.36
30_70	3.23	2.54
PLA	2.62	2.43

**FIGURE 7:**
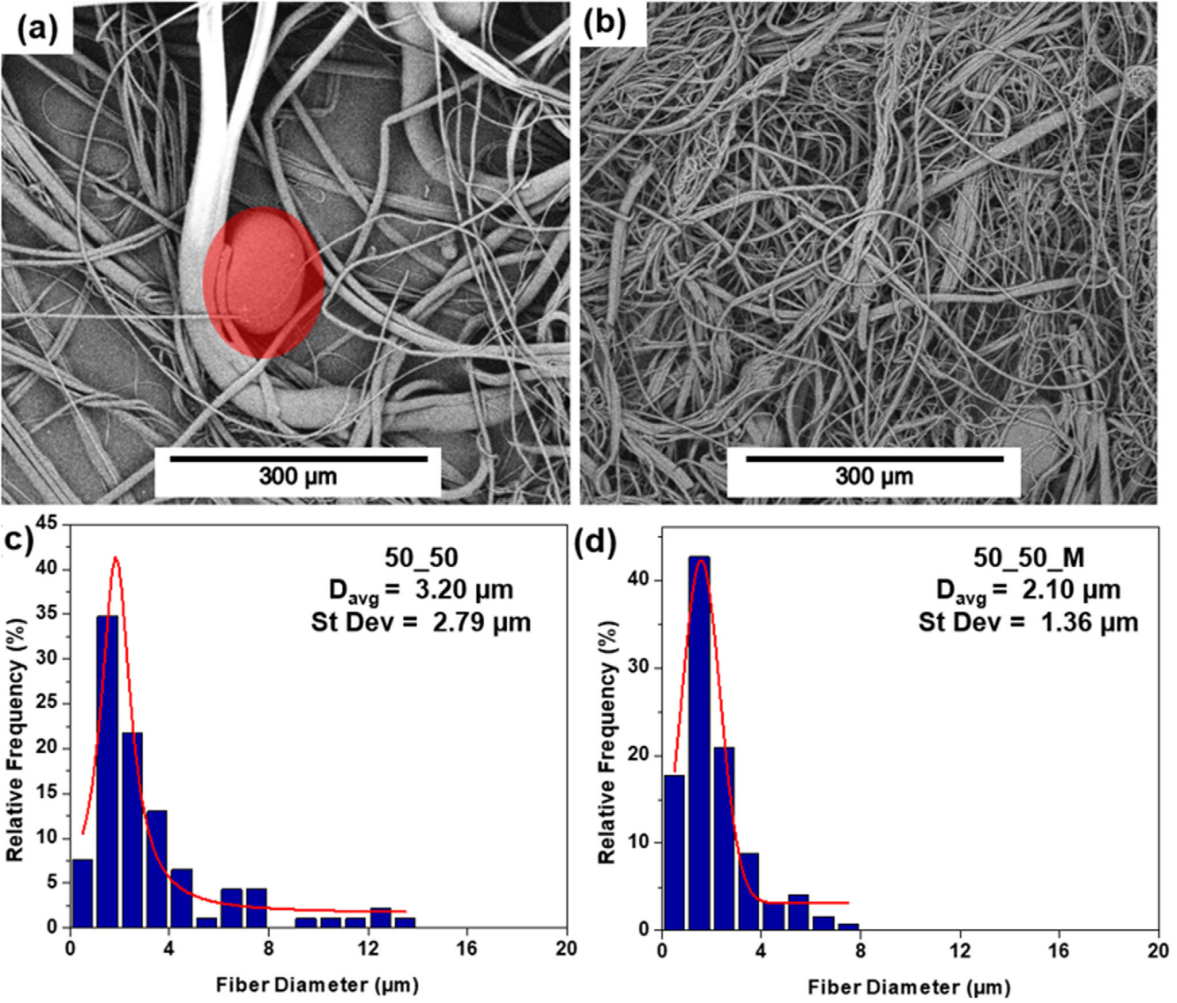
SEM image of 50–50 with beading (a) and 50‐50‐M (b) as well as their associated fiber diameter distributions (c and d).

Since phase separation is observed among the untreated blends, compatibilization is recommended to improve the end product, the effect of which can be seen in Figure 7. A clear decrease in fiber diameter and distribution is seen with the compatibilized sample. Notably, the fibers produced in this manner were even finer than either starting material alone. The finer fiber diameter is likely a result of both compatibilization and the suspected additional degradation caused by the DCP reducing the melt viscosity. Further indication of compatibilization is the limited number of droplets and shots present in the maleated sample compared to the pure blend. As previously discussed, these nonconformities likely occurred as a result of phase separation; thus, their reduced quantity suggests a more homogenous blend. In addition to the minor degree of phase separation confirmed by DSC, the presence of the remaining small number of droplets may be due to operating conditions that can be optimized to prevent this from occurring. For example, beading may occur if the operating temperature or air flow speed is too high [40]. Since the same processing conditions were applied to all samples, rather than optimized individually, it may be possible to further reduce these imperfections through changes to the operating parameters.

#### 3.3.2 Tensile Properties

**FIGURE 8:**
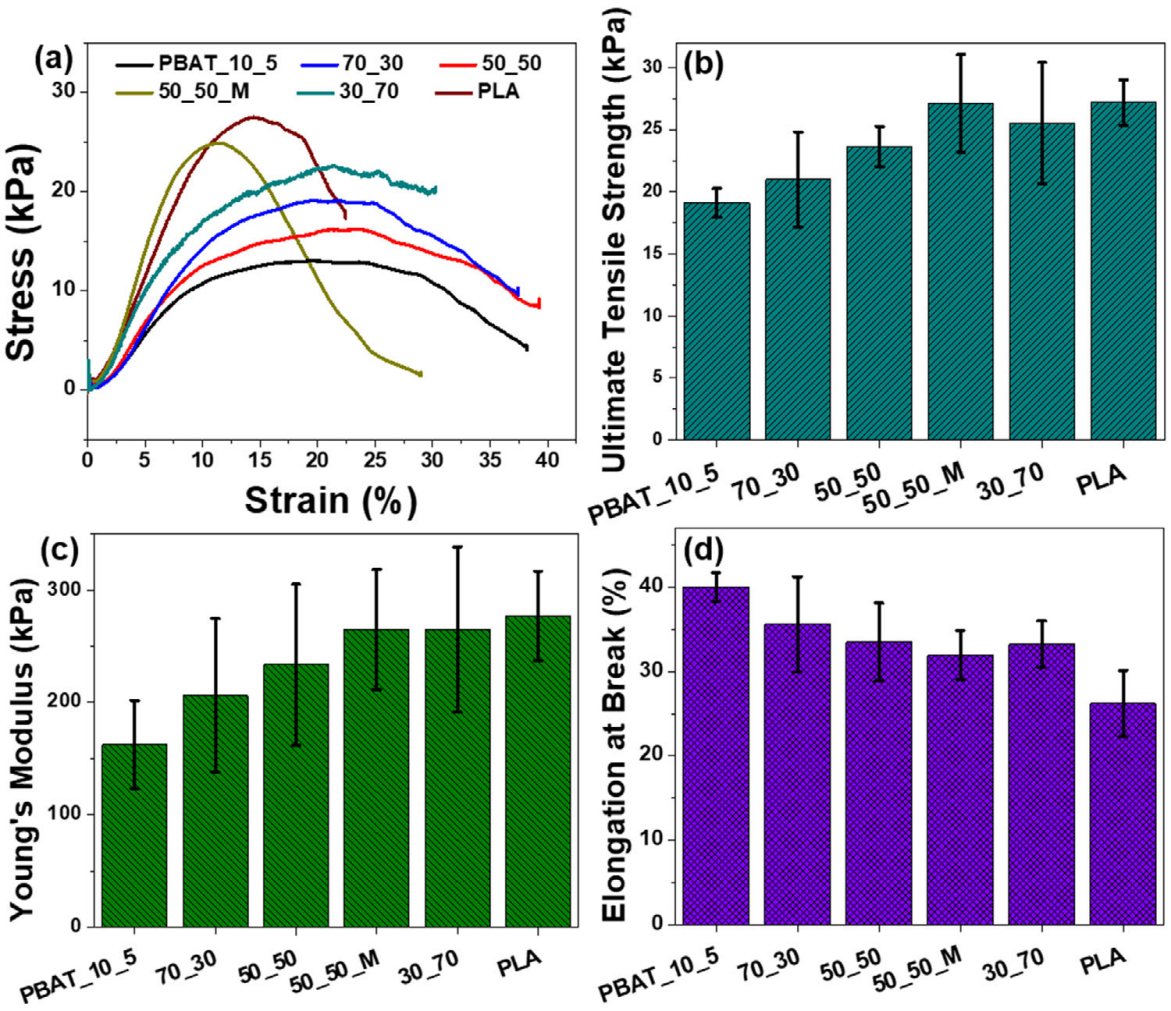
Tensile properties of PBAT/PLA nonwovens (a–d).

Tensile testing was performed on melt‐blown samples of both the starting materials and blends. Testing procedure examined ultimate tensile strength, Young's modulus, and elongation at break (Figure 8b–d). As expected, PLA performed the best in ultimate tensile strength and Young's modulus, while PBAT_10_5 proved superior for elongation at break. Performance of the blends was indicative of the ratio of the species, with increasing PLA content increasing strength and decreasing elongation. Increased tensile strength benefits the material as it makes it more durable and resistant to tearing, while improved elongation allows for more flexibility, preventing damage from repetitive deformation.

By blending PBAT and PLA, the combination gains the ability to be used in a wider range of applications where one or both of the individual materials would otherwise be incompatible. Considering the importance of both properties, a 50–50 blend appears to strike a good balance between strength and flexibility. The compatibilized blend of this ratio demonstrated a slight increase in ultimate tensile strength and Young's modulus with negligible change to elongation at break when compared to the untreated 50–50 blend. This increase in overall strength of the material is likely because the fibers are more uniform. The increased uniformity causes stress to be applied more evenly across the fabric, therefore requiring the force to overtake all of the fibers at once rather than breaking at a weak point first. This suggests that the improved compatibility of the material also improves the tensile properties as well as the fiber morphology.

## 4 Conclusions

Reactive batch mixing with aqueous hydrogen peroxide has been demonstrated to be a prominent candidate for controlled degradation of PBAT. The degradation is thought to occur via free radical‐induced chain scission as well as hydrolysis of the ester bonds. This is supported by the clear reduction of viscosity (i.e., increase in MFI) as well as the reduction in storage and loss moduli at higher peroxide concentrations. Additional support for this degradation mechanism is given by 1H NMR, detecting new peaks at 5.15 and 1.45 ppm for peroxide‐treated samples that suggest an increase in terephthalic acid and butylene fragments, respectively. It is likely that crosslinking occurs as well; however, chain scission is the dominant mechanism. Additionally, it was shown that the H_2_O_2_ treatment had little effect on the thermal and crystalline properties of the PBAT. The degraded PBAT was then melt‐blown to produce fine, uniform fibers. To reduce cost and improve the strength of the material, the degraded PBAT was blended with PLA, producing a material with a greater tensile strength than PBAT alone, as well as a longer elongation at break than PLA. The blends, however, showed evidence of immiscibility and phase separation: wide dispersity of fiber width, fiber imperfections, and inconsistent crystallization. This incompatibility was addressed using maleation, resulting in the finest fibers produced. While these fibers still exhibit small imperfections, the remaining defects can likely be eliminated through optimization of processing parameters and compatibilizer levels. Overall, this process presents an environmentally friendly method for producing high‐quality biodegradable nonwoven mats.

## Author Contributions

**Gillian Binley**: Procedure, research, formal investigation, writing, and review/ editing. **Tizazu H. Mekonnen**: Conceptualization, project management, administration, and editing.

## Conflicts of Interest

The authors declare no conflicts of interest.

## Data Availability

All data generated and/or analyzed during this study are included in the published article.
